# Supplementary material for: Polyketide synthase gene clusters in the ericoid mycorrhizal fungus Oidiodendron maius: genome-wide prediction, expression profiling, and functional characterization of knockout mutants
Source: Front Plant Sci. 2026 Sep 16;17:1924430. doi: 10.3389/fpls.2026.1924430 (PMC13623903; doi:10.3389/fpls.2026.1924430)
Supplement: Supplementary file 1 [file SupplementaryFile1.zip › Supplementary Materials/Supplementary Folder 1.zip!/Oidma1_AssemblyScaffolds_Repeatmasked/knownclusterblast/cluster58/jgi.p_Oidma1_139098_mibig_hits.html]

| MIBiG Protein | Description | MIBiG Cluster | MiBiG Product | % ID | % Coverage | BLAST Score | E-value |
| --- | --- | --- | --- | --- | --- | --- | --- |
| ABX18589.1 | nucleotide\_sugar\_dehydrogenase | BGC0000808 | Cepacian\_biosynthetic\_gene\_cluster | 34 | 93.9516129032 | 237 | 4.6e-62 |
| ADD45308.1 | nucleotide\_sugar\_dehydrogenase | BGC0000807 | Phosphonoglycans\_biosynthetic\_gene\_cluster | 31 | 98.5887096774 | 214 | 2.4e-55 |
| AAB94865.1 | UDP-glucose\_dehydrogenase | BGC0000868 | Teichuronic\_acid\_biosynthetic\_gene\_cluster | 31 | 94.5564516129 | 212 | 1.6e-54 |
| AAG26472.1 | putative\_UDP-glucose\_dehydrogenase | BGC0001411 | Polysaccharide\_B\_biosynthetic\_gene\_cluster | 32 | 94.7580645161 | 207 | 3.9e-53 |
| AAD44217.2 | GdhgA | BGC0000362 | Glycopeptidolipid\_biosynthetic\_gene\_cluster | 36 | 66.1290322581 | 180 | 3.9e-45 |
| ABM21411.1 | sugar\_biosynthesis\_protein | BGC0000766 | Exopolysaccharide\_biosynthetic\_gene\_cluster | 28 | 78.2258064516 | 130 | 4.6e-30 |
| ACY25521.1 | CcbC | BGC0000757 | Capsular\_polysaccharide\_biosynthetic\_gene\_cluster | 30 | 75.8064516129 | 129 | 1e-29 |
| ACY25516.1 | CcbC | BGC0000756 | Capsular\_polysaccharide\_biosynthetic\_gene\_cluster | 29 | 75.8064516129 | 124 | 2.5e-28 |
| BAG50498.1 | UDP-glucose\_6-dehydrogenase | BGC0000780 | O&K-antigen\_biosynthetic\_gene\_cluster | 29 | 81.25 | 123 | 9.7e-28 |
| ACY25504.1 | AcbC | BGC0000754 | Capsular\_polysaccharide\_biosynthetic\_gene\_cluster | 27 | 75.2016129032 | 120 | 4.8e-27 |
| ACY25510.1 | AcbC | BGC0000755 | Capsular\_polysaccharide\_biosynthetic\_gene\_cluster | 27 | 75.2016129032 | 120 | 4.8e-27 |
| AAK17904.1 | UDP-glucose\_dehydrogenase\_DcbC | BGC0000737 | Capsular\_polysaccharide\_biosynthetic\_gene\_cluster | 26 | 77.2177419355 | 114 | 2.6e-25 |
| AAK17922.1 | UDP-glucose\_dehydrogenase\_FcbC | BGC0000739 | Capsular\_polysaccharide\_biosynthetic\_gene\_cluster | 26 | 77.2177419355 | 114 | 4.5e-25 |
| AAV34530.1 | UDP-glucose-6-dehydrogenase | BGC0000788 | O-antigen\_biosynthetic\_gene\_cluster | 28 | 78.2258064516 | 108 | 3.2e-23 |
